# Comparative Transcriptomic Analysis of Potato and Tomato Responses to *Botrytis cinerea* Infection

**DOI:** 10.3390/plants15182815

**Published:** 2026-09-14

**Authors:** Alfonso Alvarez, Verónica Giacri, Agustina Taglioretti, Guillermo Reboledo, Inés Ponce de León, Marcos Montesano

**Affiliations:** 1 Laboratorio de Fisiología Vegetal, Facultad de Ciencias, Universidad de la República, Montevideo 11400, Uruguay; veronica.giacri@fcien.edu.uy (V.G.); ataglioretti@fcien.edu.uy (A.T.); 2 Departamento de Biología Molecular, Instituto de Investigaciones Biológicas Clemente Estable, Montevideo 11600, Uruguay; greboledo84@gmail.com (G.R.); iponce@iibce.edu.uy (I.P.d.L.)

**Keywords:** *Solanum tuberosum*, *Solanum lycopersicum*, *Botrytis cinerea*, RNAseq, defense responses, virulence

## Abstract

*Botrytis cinerea* causes gray mold disease in numerous plant species, including economically important solanaceous crops. To investigate the molecular interaction between *B. cinerea* and *Solanum tuberosum*, we performed dual RNA-seq analyses of infected potato leaves at 24 and 72 h post inoculation (hpi). A total of 5932 potato genes were differentially expressed during infection, including genes associated with pathogen perception, hormone signaling, transcriptional regulation, and defense responses to biotic stress. In parallel, 2162 fungal genes showed differential expression, including genes encoding plant cell-wall-degrading enzymes, reactive oxygen species-related proteins, detoxification enzymes, toxins, and candidate secreted effectors. Comparative analyses with an independent transcriptomic dataset from tomato infected with *B. cinerea* revealed both shared and distinct transcriptional patterns. Similar expression patterns were observed for genes associated with jasmonic acid, ethylene signaling, and stress responses, while differences were found in the induction of genes involved in specialized metabolism. Potato exhibited increased expression of genes associated with germacrene B and α-farnesene biosynthesis, whereas tomato showed induction of genes related to viridiflorene and costunolide production. In addition, fungal genes involved in botrydial biosynthesis and several secreted virulence-associated enzymes showed stronger induction during tomato infection. These findings provide new insights into the transcriptional responses of potato to *B. cinerea* infection and the similarities and differences observed in comparison with tomato.

## 1. Introduction

Plants are susceptible to diseases caused by phytopathogens such as bacteria, fungi, oomycetes, nematodes, and viruses, which could negatively affect plant growth and cause large economic losses in different crops [1]. However, plants exhibit physical and chemical barriers against pathogen invasion, such as the plant cell wall and cuticle, and constitutively produced antimicrobial compounds [2]. In addition, plants can induce biochemical responses and mechanisms to recognize phytopathogens, including pattern recognition receptors (PRRs) capable of recognizing a series of pathogen-associated molecular patterns (PAMPs) or damage-associated molecular patterns (DAMPs), leading to the activation of PAMP-triggered immunity (PTI) [3]. Moreover, intracellular receptors, mainly nucleotide-binding leucine-rich repeat immune receptors (NLRs), directly or indirectly recognize pathogen effectors that suppress the immune response and induce effector-triggered immunity (ETI) [3]. Recent studies have revealed that PTI and ETI work together to potentiate the immune response, leading to inhibition of pathogen growth and, in some cases, to resistance [4]. PTI and ETI activation triggers multiple defense responses, including changes in ion fluxes across the plant plasma membrane, the production of reactive oxygen species (ROS), activation of mitogen-activated protein kinase (MAPK) signaling pathways, regulation of plant hormones, expression of pathogenesis-related (PR) proteins, production of antimicrobial compounds, and cell-wall reinforcement at infection sites. Additionally, ETI specifically induces localized cell death known as the hypersensitive response (HR) [5].

Solanaceae is a highly diverse family of plants, comprising 3000–4000 species classified into approximately 90 genera. Many members of this family are crucial food resources and have become key subjects of genetic research, including potato (*Solanum tuberosum*) and tomato (*Solanum lycopersicum*) [6]. Both species are native to the Americas and share over 90% genomic similarity despite structural differences [7]. *S. tuberosum* has over 4000 edible varieties, and the production of its tubers is crucial for human consumption, with global production exceeding 380 million tons in 2023 [8,9]. The fruit of *S. lycopersicum* is one of the most consumed vegetables worldwide [10], being one of the main horticultural products with significant economic impact, and its global production exceeded 190 million tons during the year 2023 [9]. Both solanaceous plants are susceptible to multiple diseases affecting various organs, leading to significant reductions in production during cultivation and postharvest stages.

*Botrytis cinerea* is the etiological agent of gray mold disease, a necrotrophic fungus responsible for significant annual economic losses [11]. The interaction between this fungus and the host plant begins when spores land on the host surface or enter plant tissues through wounds caused by mechanical damage produced by wind, rain, herbivores, and other means, allowing spores to germinate and cause plant disease. [12]. *B. cinerea* secretes enzymes such as cutinases and lipases, which degrade the cuticle and facilitate fungal entry, as well as plant cell-wall-degrading enzymes (PCWDEs), including pectinases and polygalacturonases [12]. Additionally, this fungus produces phytotoxic compounds and oxalic acid (OA) and induces ROS accumulation, leading to host cell death [13]. These mechanisms result in rapid tissue maceration, enabling *B. cinerea* to sporulate and produce inoculum for further infections [12]. Gray mold is particularly common in solanaceous crops. In tomato, gray mold is prevalent in greenhouse-grown plants, affecting various plant organs and causing postharvest damage [14]. The interaction between *B. cinerea* and *S. lycopersicum* has been extensively studied, and transcriptome profiling data has been generated [15,16]. Although gray mold is not one of the primary diseases affecting *S. tuberosum*, it has been reported to cause significant damage to potato crops in the USA [17]. Furthermore, the potato–*B. cinerea* interaction has been proposed as an interesting model system to study fungal diseases [18]. However, to date, no transcriptomic profiling of this interaction has been reported. Comparative transcriptomic analyses between related host species can provide insights into conserved defense mechanisms, as well as contrasting transcriptional patterns associated with pathogen colonization strategies. Here, we performed a dual RNA-seq analysis of potato leaves infected with *B. cinerea* at two early time points to characterize host transcriptional responses and fungal virulence strategies. In addition, we compared potato transcriptomic responses with previously published tomato–*B. cinerea* transcriptomic datasets [16] to identify conserved and contrasting defense-related transcriptional patterns in these two Solanaceae species.

## 2. Results

### 2.1. Disease Symptoms and Fungal Colonization in S. tuberosum Infected with B. cinerea

**Figure 1:**
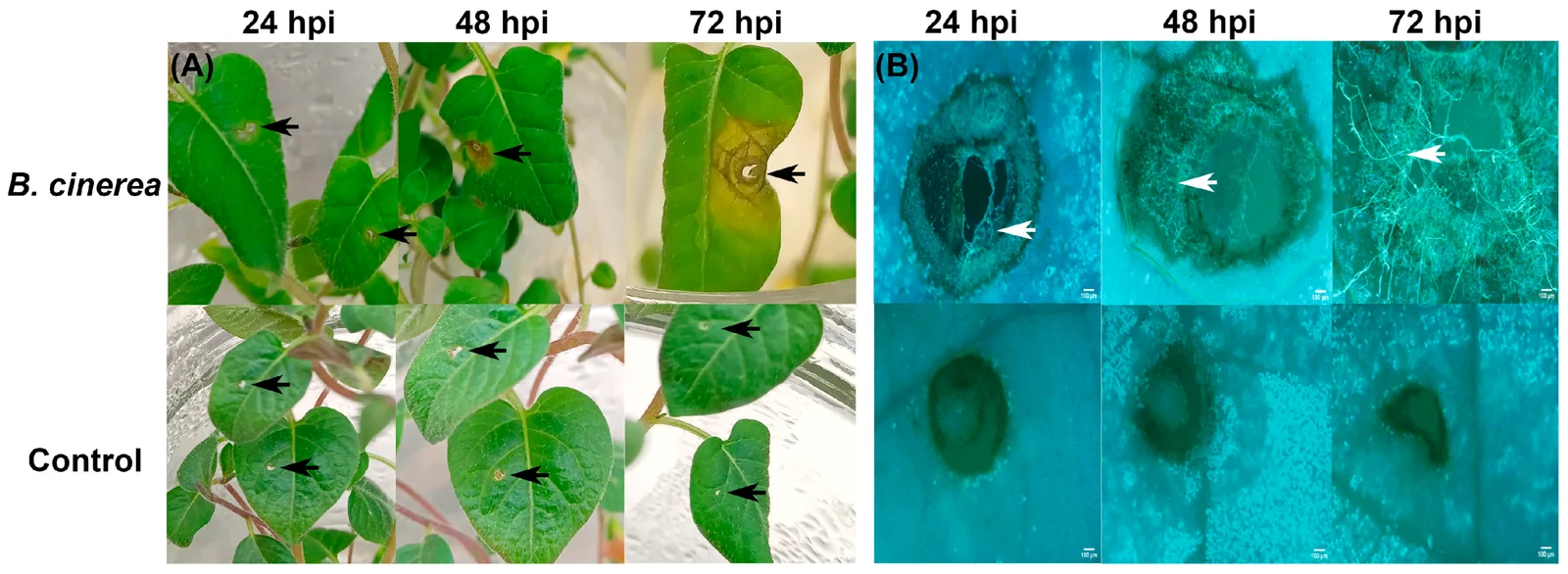
Representative symptoms and fungal colonization in *S. tuberosum* leaves after *B. cinerea* inoculation. (**A**) Representative symptoms in potato leaves inoculated with *B. cinerea* spores at 24, 48 and 72 hpi. Black arrows indicate the inoculation site; water-treated leaves were used as controls. The leaves shown in panel A were subsequently stained for microscopic visualization of fungal hyphae with fluorescent dye solopenil flavine 7GFE500 (**B**), illustrating fungal colonization in the corresponding potato leaves at 24, 48 and 72 hpi. White arrows indicate *B. cinerea* hyphal development. The scale bar represents 100 µm.

Disease symptoms on *S. tuberosum* leaves inoculated with *B. cinerea* spores were visible at 24 h post inoculation (hpi), with initial browning at the inoculation site. Representative leaves collected at 48 and 72 hpi showed more extensive tissue maceration and chlorosis than the representative leaves collected at 24 hpi (Figure 1A). In contrast, control leaves showed small scars caused by mechanical inoculation injury; however, they remained healthy and preserved their normal green coloration at all time points. To further investigate the *B. cinerea* infection process, solophenyl flavine staining was employed. At 24 hpi, spore germination and early hyphal growth were detected, while leaf samples collected at 48 and 72 hpi showed more extensive hyphal colonization (Figure 1B). As expected, no fungal structures were detected in the control leaves. These observations confirm successful colonization of potato leaves by *B. cinerea* at the analyzed time points.

### 2.2. Identification of DEGs and Biological Processes Involved in S. tuberosum Defense Activation During B. cinerea Infection

**Figure 2:**
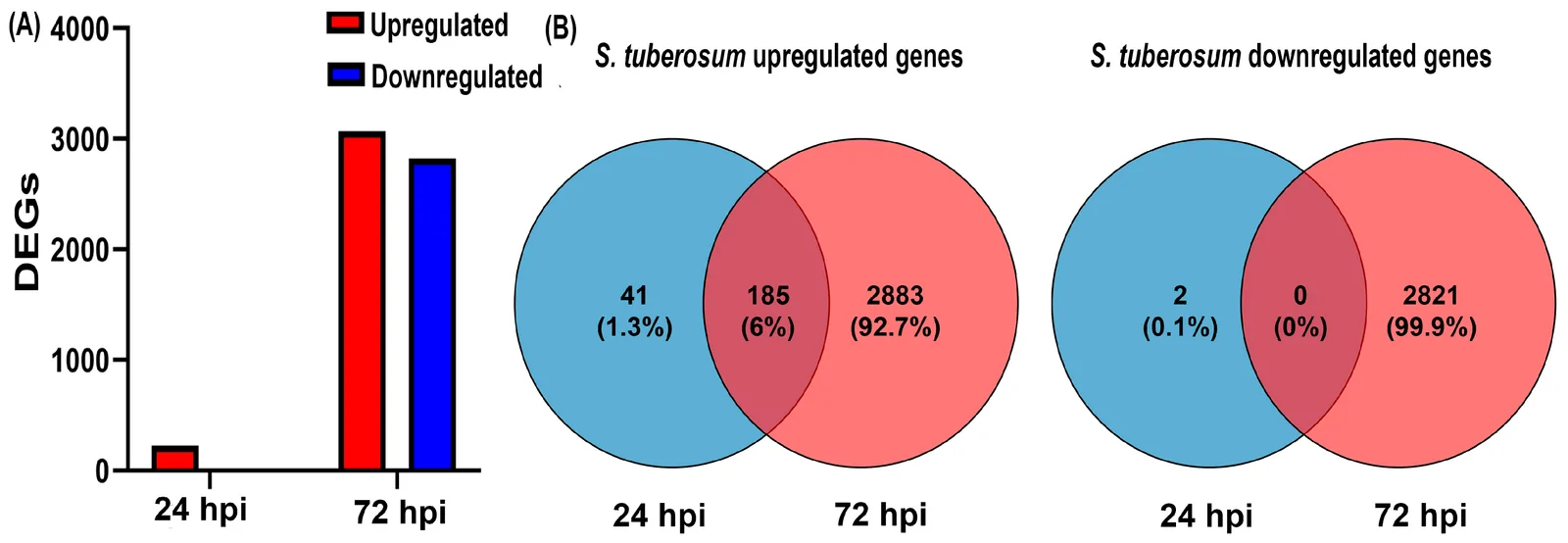
*S. tuberosum* DEGs identified in response to *B. cinerea* infection. (**A**) Number of potato DEGs at 24 and 72 hpi compared with water-treated controls. (**B**) Venn diagrams showing overlaps of potato DEGs at 24 and 72 hpi.

To identify the defense mechanisms activated in *S. tuberosum* during early stages of *B. cinerea* infection (24 and 72 hpi), RNA sequencing was performed on infected and mock-treated potato leaves. Reads mapped successfully to the *S. tuberosum* genome, with mapping rates ranging from 62.5% to 80.7% (Table S2). A total of 5932 differentially expressed genes (DEGs) were identified, representing approximately 18% of the 32,917 annotated potato gene models. Marked temporal differences were observed in the transcriptional response to *B. cinerea*: at 24 hpi, 226 genes were upregulated and only two were downregulated, whereas at 72 hpi, 3068 genes were induced and 2821 were repressed (Table S3, Figure 2A). The stronger transcriptional reprogramming observed at 72 hpi coincided with more extensive symptoms and increased fungal colonization. Among these, 185 genes were upregulated at both time points (Figure 2B). GO enrichment analysis revealed no significantly enriched biological processes at 24 hpi, consistent with the low number of DEGs detected at this stage. In contrast, multiple defense-related processes were significantly enriched at 72 hpi, including chitin catabolism, cell-wall macromolecule metabolism, regulation of jasmonic acid-mediated signaling, and phenylpropanoid biosynthesis. Several biological processes associated with photosynthesis, such as light harvesting, electron transport, and chlorophyll biosynthesis, were strongly downregulated at 72 hpi (Table S4). These results indicate that defense-related biological processes are activated at 72 hpi, while fungal infection concurrently suppressed photosynthetic functions.

### 2.3. S. tuberosum Activates Pathogen Perception, Hormonal Pathways, and Other Defense Mechanisms During B. cinerea Infection

RNA-seq analysis revealed differential expression of at least 260 *S. tuberosum* genes encoding receptor-like kinases (RLKs) and associated proteins at 24 and 72 hpi in response to *B. cinerea* infection (Table S3). At 24 hpi, six RLKs with diverse extracellular domains were upregulated, including one lectin-RLK, one leucine-rich repeat (LRR)-RLK, and four wall-associated kinase (WAK)-RLKs, which remained induced at 72 hpi. Additionally, one D-mannose binding lectin and three LRR proteins, including an LRR-disease resistance protein RPP13-related (NLR), were detected at 24 hpi. At 72 hpi, a significantly higher number of RLK and associated protein genes showed differential expression, including BAK1; chitin elicitor receptor kinase (CERK1); cysteine-rich receptor kinases (CRKs); and LysM-, lectin-, WAK-, and LRR-RLKs. In total, 121 RLK-related genes were upregulated, and 137 were downregulated. Genes involved in calcium and MAPK signaling, ROS production, and key transcription factors (WRKY, AP2/ERF, MYB, HLH, GRAS, HLZ, MADS-box, and HSF) were also differentially expressed. Hormone-related genes were mainly induced at 72 hpi, particularly those involved in jasmonic acid (JA) and ethylene (ET) biosynthesis and signaling. In the JA pathway, several genes encoding key biosynthetic enzymes were upregulated, including lipoxygenases (LOXs), allene oxide synthase (AOS), and 12-oxophytodienoic acid reductase 3 (OPR3), as well as genes encoding JA signaling components such as TIFY domain-containing proteins and Jasmonate ZIM-domain (JAZ) proteins. Genes related to the ET pathway were also induced, including those encoding ethylene-responsive transcription factors (ERFs); ethylene receptors (ETRs); and enzymes involved in ET biosynthesis, such as 1-aminocyclopropane-1-carboxylate synthase (ACS) and 1-aminocyclopropane-1-carboxylate oxidase (ACO) (Table S3). Salicylic acid (SA)-related components were also differentially expressed, including phenylalanine ammonia-lyase (PAL) and salicylic acid glucosyltransferase (SGT), which catalyzes the formation of SA O-β-glucoside. Genes associated with auxin (AUX), cytokinin (CK), gibberellin (GA), abscisic acid (ABA), and brassinosteroid pathways also showed differential expression (Table S3). Furthermore, genes related to the production of PR proteins and antimicrobial compounds, phenylpropanoid biosynthesis, cell-wall reinforcement at infection sites, and hypersensitive response (HR) were modulated during infection. At 24 hpi, some DEGs encoding PRs (including β-1,3-glucanase, chitinase, and Bet v I), enzymes involved in phytoalexins, terpenoids, and alkaloids biosynthesis, as well as proteins implicated in the modification of cell-wall components, were upregulated in response to *B. cinerea* infection. Accordingly, at 72 hpi, the number of upregulated plant DEGs associated with these defense responses increased, and genes involved in phenylpropanoid biosynthesis and HR-related proteins were also induced (Table S3). Together, these results indicate broad transcriptional changes in genes associated with pathogen perception, signaling, hormone-mediated pathways, and defense responses in potato during *B. cinerea* infection.

### 2.4. Comparative Analysis of Defense-Related DEGs in Potato and Tomato During Infection with B. cinerea

To compare transcriptional responses in closely related Solanaceous hosts, previously published RNA-seq data from *S. lycopersicum* infected with *B. cinerea* spores were reanalyzed [16], focusing on the closest available time points (23 and 47 hpi) to those used in our analysis of *S. tuberosum* (24 and 72 hpi, respectively). Although these sampling points were close in time, they may not represent identical stages of infection. Table S2 summarizes the number of reads mapped to the *S. lycopersicum* reference genome for each analyzed sample. A total of 3192 upregulated and 3386 downregulated genes were identified at 23 hpi, and 2906 upregulated and 4344 downregulated genes were identified at 47 hpi (Table S5). When comparing the time points close to 24 hpi, *S. lycopersicum* exhibited a higher number of DEGs than *S. tuberosum*. At the other analyzed time points (47 hpi in tomato vs. 72 hpi in potato), the number of DEGs was more comparable between the two species. Of the total DEGs identified in *S. lycopersicum*, 5538 had orthologs among the DEGs previously described in *S. tuberosum* in response to *B. cinerea* infection (Table S6). GO enrichment analysis revealed several shared upregulated biological processes, including chitin catabolism, phenylpropanoid biosynthesis, and JA-related responses. In contrast, tomato showed exclusive enrichment of SA-related and HR-associated processes (Table S7), while HR-associated genes were downregulated in potato at 72 hpi. Tomato also displayed strong repression of photosynthesis-related processes following infection.

**Figure 3:**
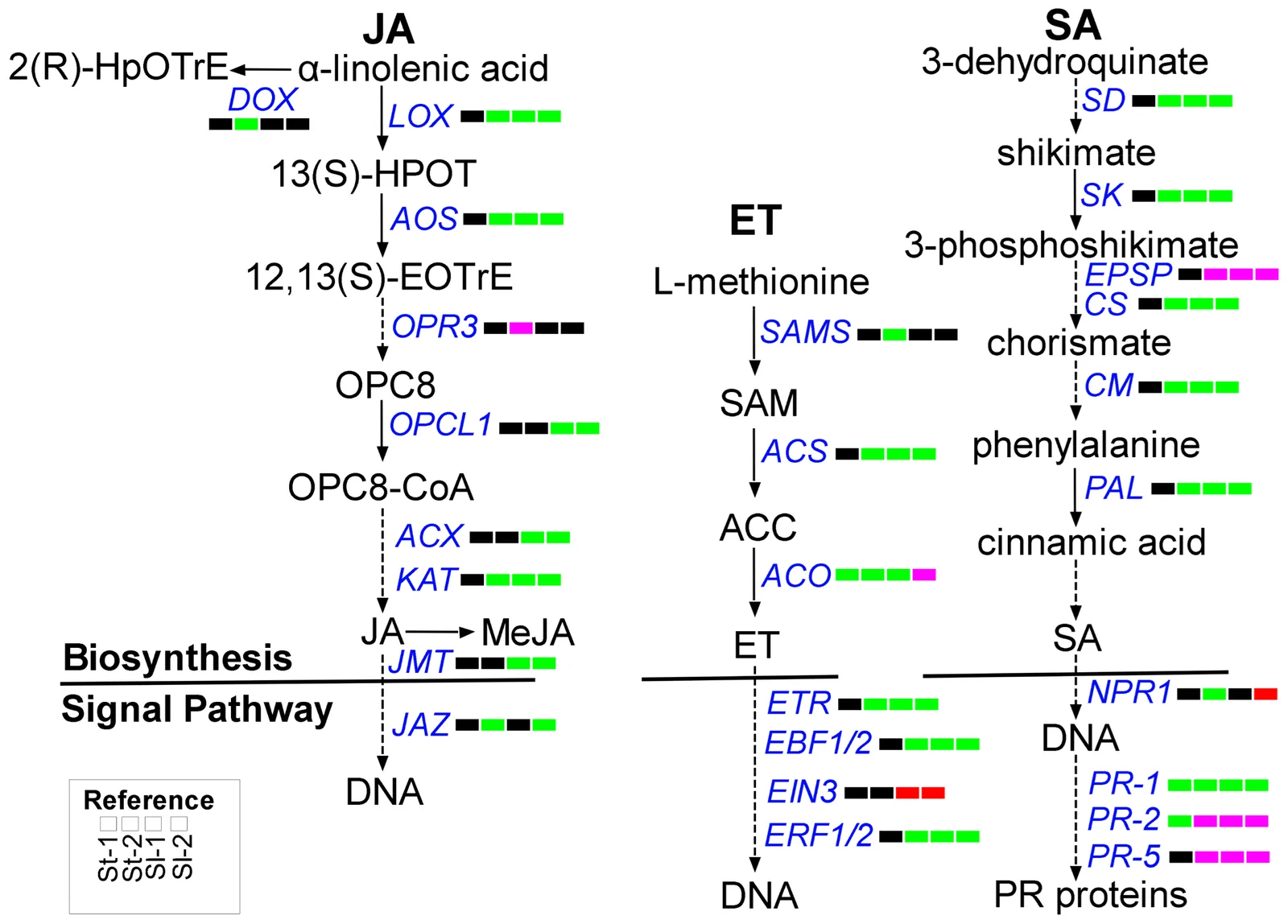
*S. tuberosum* and *S. lycopersicum* DEGs related to hormonal biosynthesis and signaling pathways identified in response to *B. cinerea* infection. Schematic representation of JA, ET and SA biosynthesis and signaling pathways. Solid arrows indicate direct links between metabolites, while dotted arrows represent indirect associations with the indicated metabolite. Transcript expression relative to the water control is indicated by colored squares for each gene: green squares represent significant induction, red squares represent significant repression, black squares indicate non-significant changes, and pink squares indicate that different members of the same gene family showed opposite expression patterns within the corresponding comparison, with some genes significantly induced and others significantly repressed St-1, 24 hpi; St-2, 72 hpi; Sl-1, 23 hpi; Sl-2,47 hpi.

Consistent with patterns observed in potato, tomato exhibited a broad transcriptional activation of genes associated with pathogen perception and signaling. Both species showed highly conserved expression profiles for genes associated with early immune responses, including those encoding pattern recognition receptors (e.g., BAK1, CERK1, LysM-containing receptors, lectin receptor kinases, WAKs, CRKs, and LRR-containing proteins), as well as components of calcium signaling, MAPK cascades, and ROS production. Expression patterns were also largely conserved for several transcription-factor families (e.g., WRKY, MYB, and AP2/ERF). Similarly, both species displayed broadly conserved expression profiles for genes related to cytokinin, auxin, ABA, and gibberellin biosynthesis and signaling. However, specific differences emerged in the expression of genes related to ET, JA, and SA, as well as oxylipin metabolism (Figure 3). Potato showed exclusive induction of α-dioxygenase 1 (α-DOX1) at 72 hpi, whereas no differential expression of this gene was detected in tomato. In addition, potato specifically induced 12-oxophytodienoic acid reductase 3 (OPR3), a key enzyme of the jasmonic acid biosynthetic pathway. In the ET pathway, S-adenosylmethionine synthase (SAMs) was induced only in potato at 72 hpi, while ethylene-insensitive protein (EIN), a key signaling component, was repressed only in tomato. For the SA pathway, nonexpressor of pathogenesis-related genes 1 (NPR1), a central regulator of SA signaling, was induced in potato at 72 hpi but repressed in tomato at 47 hpi, suggesting differences in the regulation of early hormonal signaling between the analyzed datasets. Both species also shared conserved transcriptional activation of general defense mechanisms, including genes involved in cell-wall reinforcement (e.g., peroxidases, cellulose synthases, xyloglucan endotransglucosylases, and laccases); oxidative stress responses (e.g., catalase and glutathione S-transferases); and PR proteins such as PR1, PR2 (β-1,3-glucanases), PR3 (chitinases), PR5 (thaumatin), and PR10 (Bet v I). These shared patterns indicate common defense-related responses activated during *B. cinerea* infection in both datasets.

**Figure 4:**
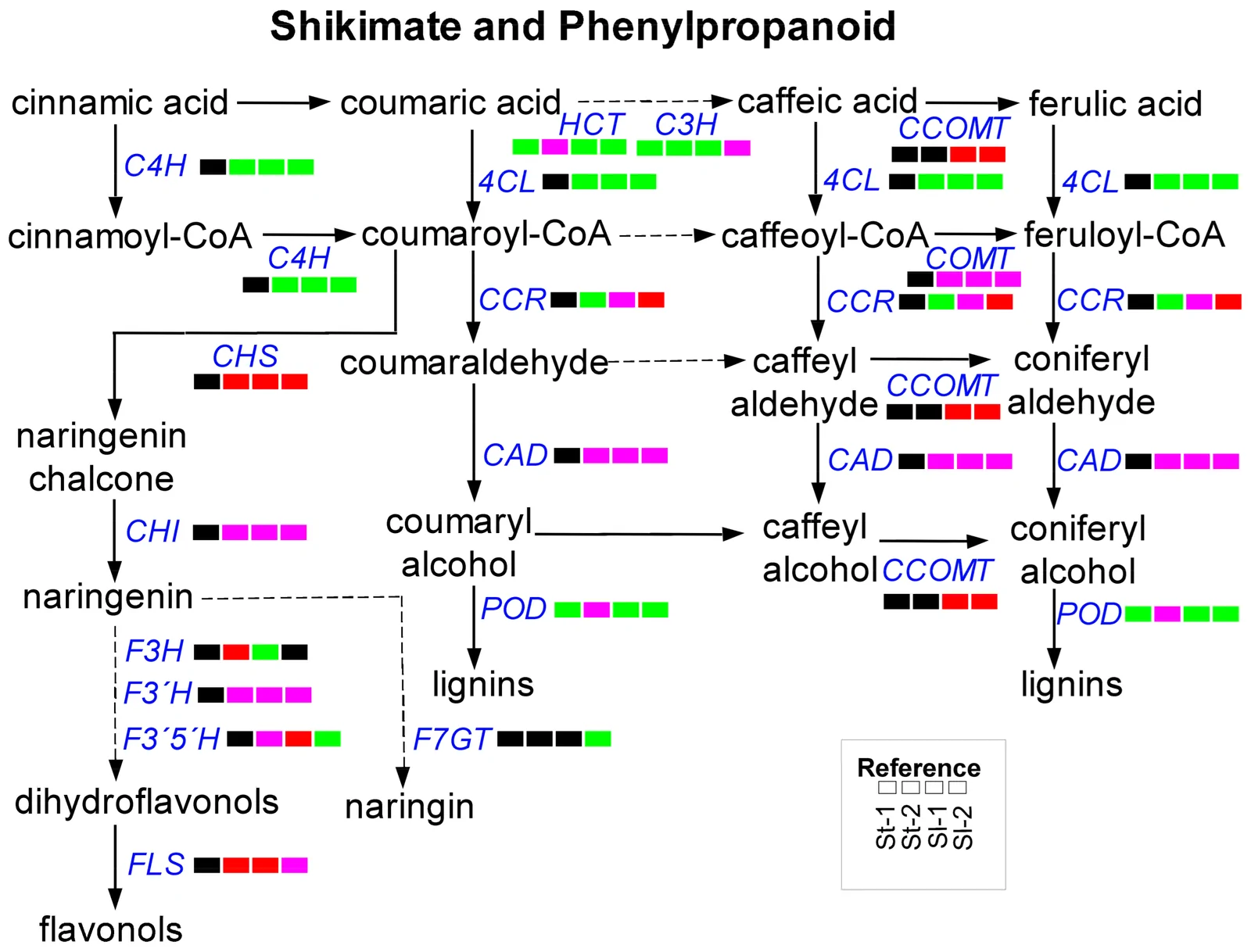
*S. tuberosum* and *S. lycopersicum* DEGs related to shikimate and phenylpropanoid biosynthesis identified in response to *B. cinerea* infection. Schematic representation of shikimate and phenylpropanoid biosynthesis pathways. Color coding, arrow styles, and time-point designations are as described in Figure 3.

However, subtle differences between the analyzed datasets were observed in the expression of genes related to the biosynthesis of specialized antimicrobial compounds, particularly phenylpropanoids, sesquiterpenoids, and monoterpenoids. When examining secondary metabolism, both species showed broadly similar expression patterns of genes associated with major biosynthetic pathways. Differences between potato and tomato datasets were observed in particular branches. In the phenylpropanoid pathway (Figure 4), both potato and tomato showed differential expression of several core biosynthetic genes, but subtle variations were noted in the regulation of certain flavonoid-related genes, such as flavanone 3-hydroxylase (F3H), flavonol synthase (FLS), and flavanone 7-O-β-glucosyltransferase (F7GT), which displayed species- and time-dependent expression dynamics.

**Figure 5:**
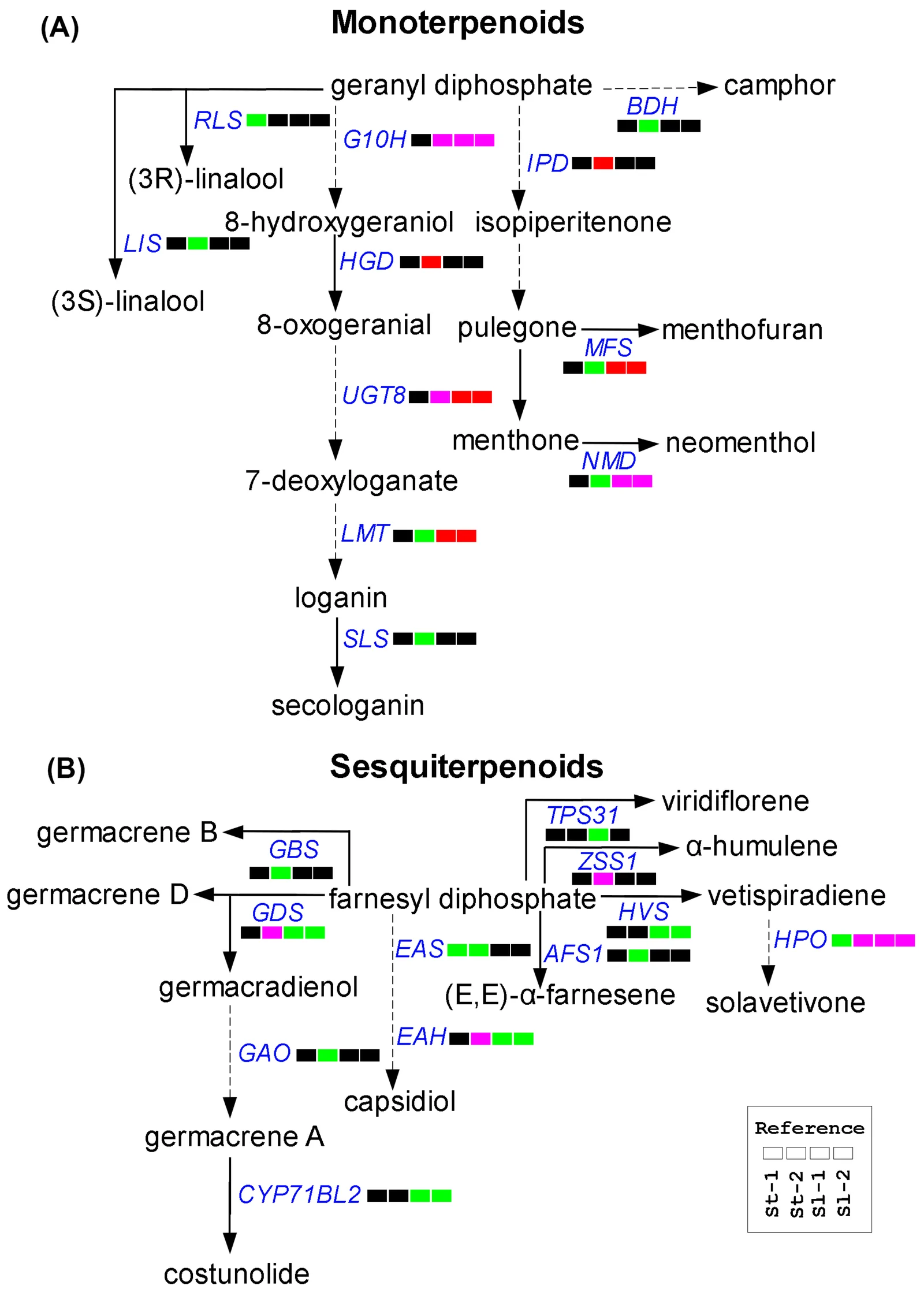
*S. tuberosum* and *S. lycopersicum* DEGs related to monoterpenoid and sesquiterpenoid biosynthesis identified in response to *B. cinerea* infection. (**A**) Monoterpenoid biosynthesis pathway. (**B**) Sesquiterpenoid biosynthesis pathway. Color coding, arrow styles, and time-point designations are as described in Figure 3.

In the monoterpenoid pathway, several genes associated with linalool, camphor, loganin, secologanin, and menthofuran biosynthesis were induced exclusively in potato, while menthofuran-associated genes were repressed in tomato (Figure 5A). In the sesquiterpenoid pathway, genes related to germacrene B, α-farnesene, and germacrene A acid production were specifically upregulated in potato, whereas genes associated with viridiflorene, vetispiradiene, and costunolide biosynthesis were differentially expressed only in tomato (Figure 5B). In contrast, genes associated with isoquinoline alkaloid biosynthesis showed no major differences between the two species (Supplementary Figure S1). These findings indicate generally similar expression patterns of genes associated with secondary metabolism in both datasets, while differences between potato and tomato were observed in specific antimicrobial biosynthetic pathways.

### 2.5. B. cinerea Transcriptional Profiles and Virulence Factors During S. tuberosum Infection

**Figure 6:**
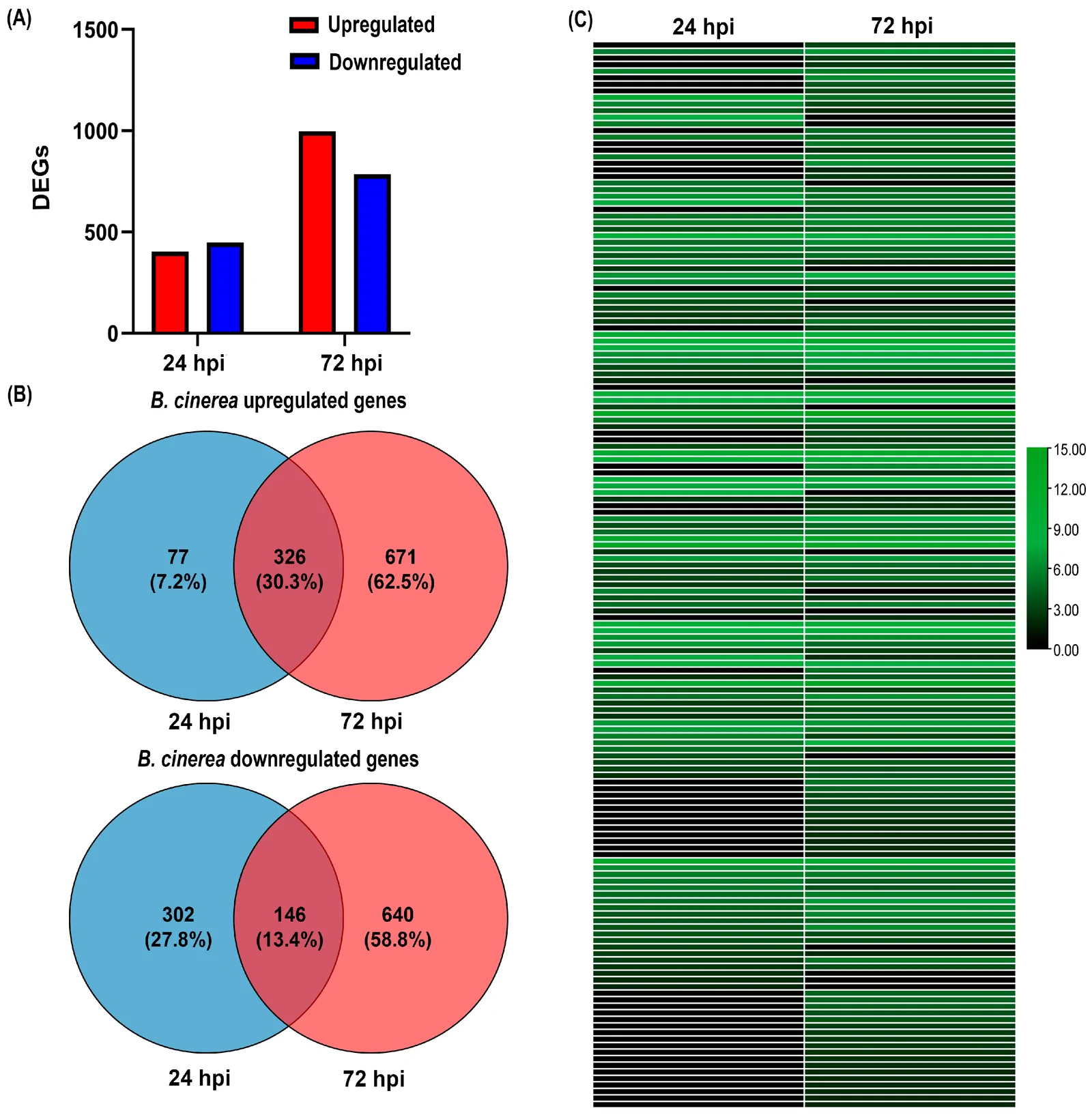
*B. cinerea* DEGs identified during *S. tuberosum* infection. (**A**) Number of fungal DEGs in potato leaves inoculated with *B. cinerea* compared with PDA control. (**B**) Venn diagrams of fungal DEGs at 24 and 72 hpi. (**C**) DEGs encoding secreted *B. cinerea* proteins induced during infection of potato. Only DEG values with log2 FC ≥ 2 and FDR < 0.05 were considered for heatmap construction for 24 and 72 hpi. Normalized values relative to control are shown on a green scale. DEGs are grouped by description.

To identify fungal genes involved during *S. tuberosum* infection, we analyzed the transcriptional profiles at 24 and 72 hpi. Among all samples, up to 87.2% of the reads in the libraries successfully mapped to the reference genome of *B. cinerea* (Table S8) and 2162 fungal DEGs were identified, with 1074 upregulated and 1088 downregulated genes (Figure 6A). The number of upregulated *B. cinerea* genes increased between the analyzed times—403 at 24 hpi to 997 at 72 hpi—while 30% of upregulated genes were identified at both times. A similar pattern was observed in downregulated genes: 448 were identified at 24 hpi and 786 at 72 hpi, with 13% of these genes identified at both times (Figure 6B, Table S9). Gene ontology term enrichment analysis was performed to identify the fungal biological processes involved in *S. tuberosum* infection (Table S10). At 24 and 72 hpi, the number of enriched upregulated processes exceeded those that were downregulated. Enriched upregulated biological processes were related to the catabolism of different cellular elements, including polysaccharides, carbohydrates, cellulose, xylans, cellular lipids, arabinans, α-amino acids, glutamines, and fatty acids. Additionally, the extracellular region was the most significantly upregulated cellular component at both times. In contrast, enriched downregulated biological processes included terms related to the (1-3)-β-D-glucan metabolic process at 24 hpi and metal ion transport at 72 hpi.

Upregulated DEGs encoding proteins associated with the *B. cinerea* secretome were identified by comparing our RNAseq data with previously published secretomes [19,20]. Consistent with infection progression, the number of upregulated genes encoding secreted enzymes increased over the course of infection; 108 genes were induced at 24 hpi, and 146 were induced at 72 hpi (Figure 6C, Table S11). At both time points, we identified genes encoding enzymes involved in the degradation of cutin and cell-wall components including pectin, cellulose, and hemicellulose. Most genes showed comparable expression levels between the two time points, although several were expressed more strongly at 24 hpi. Genes encoding secreted enzymes acting on carbohydrates and side chains of the plant cell wall were also upregulated during *S. tuberosum* infection, with expression levels similar at 24 and 72 hpi, while enzymes acting on mannan were only induced at 72 hpi. In addition, several genes encoding hydrolases and peptidases were induced at both time points. Other *B. cinerea* genes encoding virulence factors were upregulated in potato leaf tissues, including necrosis- and ethylene-inducing proteins (Bcnep1 and Bcnep2), common fungal extracellular membrane (CFEM) domain-containing proteins, and Bcmp1 (Deuterolysin). Furthermore, 55 candidate secreted effectors (51 apoplastic and four cytoplasmic) were identified among the upregulated fungal DEGs (Table S11), including α-amylase, pectin lyases, Bcnep1, Bcnep2, polygalacturonases, glucanases, peptidases, cutinases, and several uncharacterized proteins. Overall, a wide range of genes encoding secreted enzymes, effectors, and virulence-associated proteins were upregulated during potato infection.

### 2.6. Comparative Analysis of B. cinerea DEGs During Infection of S. tuberosum and S. lycopersicum

**Figure 7:**
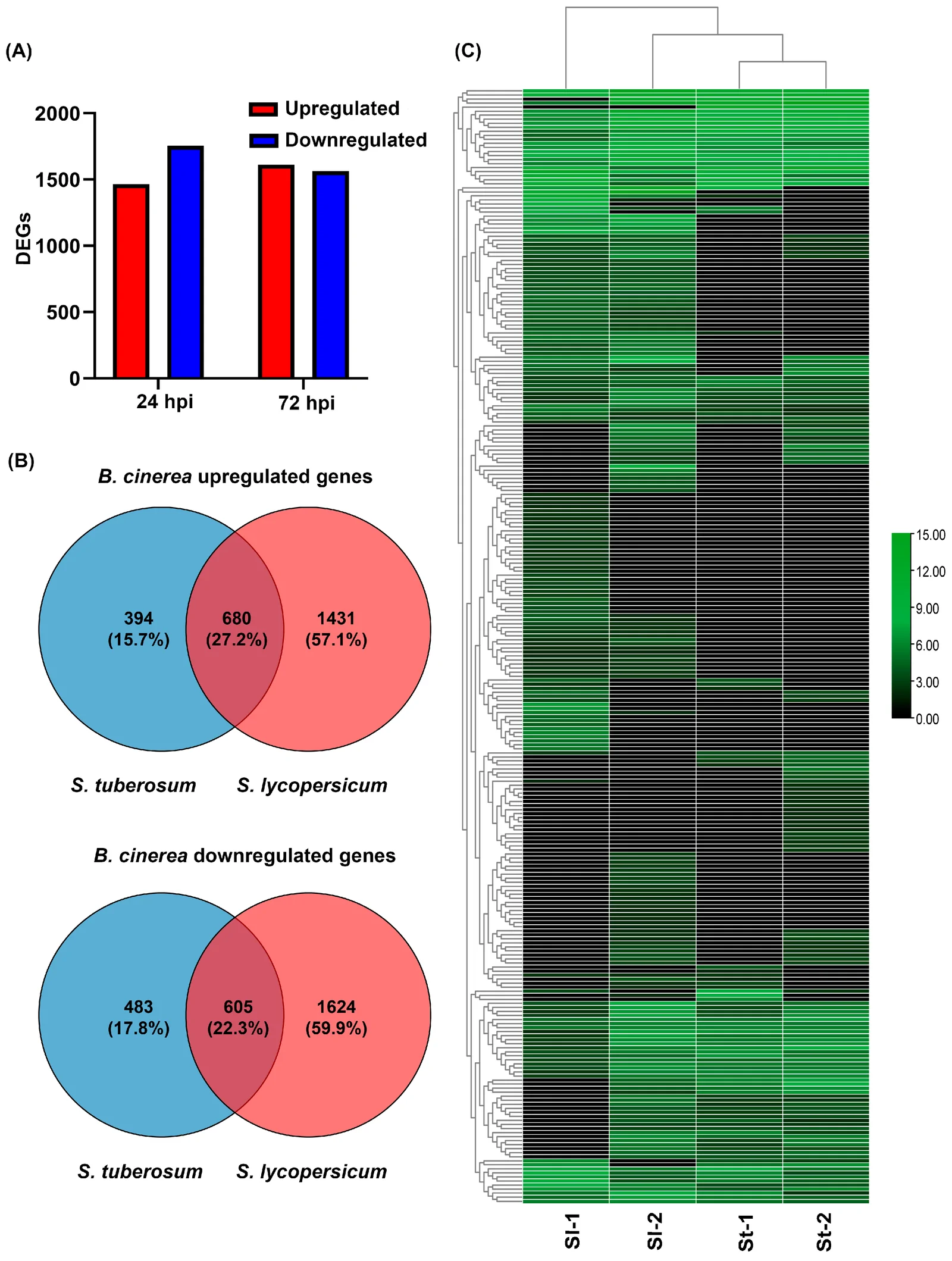
Comparative analysis of *B. cinerea* DEGs identified during infection of *S. tuberosum* and *S. lycopersicum*. (**A**) Number of fungal DEGs in tomato leaves inoculated with *B. cinerea* compared with PDA control. (**B**) Venn diagrams showing overlap of fungal DEGs in potato (merging 24 and 72 hpi) and tomato (merging 23 and 47 hpi). (**C**) Hierarchical clustering of fungal DEGs encoding secreted proteins during infection of potato (24 and 72 hpi) and tomato (23 and 47 hpi). Criteria for DEG selection and color scale are as described in Figure 7. St-1, 24 hpi; St-2, 72 hpi; Sl-1, 23 hpi; Sl-2, 47 hpi.

To compare the expression patterns of fungal DEGs involved in the infection process of *B. cinerea* in another solanaceous plant, the same RNA-seq datasets previously used for the host comparative analysis were reanalyzed, selecting tomato samples infected with *B. cinerea* spores at 23 and 47 hpi, which represented the closest available sampling points to the potato infection stages analyzed in this study. Information related to the number of reads mapped to the *B. cinerea* reference genome from each sample is provided in Table S12. The number of fungal DEGs identified in tomato was higher than those detected in our study, although differences in experimental conditions and infection stages should be considered. In tomato, there were 1464 upregulated and 1754 downregulated fungal genes at 23 hpi and 1611 upregulated and 1563 downregulated genes at 47 hpi (Figure 7A, Table S12). Thus, the tomato dataset showed a higher number of fungal DEGs at the analyzed sampling points, which may reflect differences in fungal development, infection progression, or experimental conditions. Considering only those fungal DEGs induced in both hosts, 146 genes were upregulated at all time points, while 534 genes were induced for at least one time point in each host. Specifically, 394 and 1431 fungal genes were exclusively upregulated in potato and tomato, respectively (Figure 7B, Table S13).

Among the genes induced only in potato, a high proportion encoded uncharacterized proteins, while others encoded proteins such as β-mannosidase, β-lactamase domain-containing protein, endo-β-glucanase, and ricin B-type lectin domain-containing protein. Conversely, fungal DEGs induced only in tomato were mostly induced at both time points and included genes encoding bZIP domain-containing protein, chalcone isomerase domain-containing protein, chorismate mutase, chitin-binding type-4 domain-containing protein, glutathione peroxidase, glutathione reductase, isopentenyl-diphosphate Delta-isomerase, MAPK kinase, myb-like domain-containing protein, peroxins, and uncharacterized proteins. Interestingly, fungal genes involved in botrydial biosynthesis (Bcbot1, Bcbot2, Bcbot3, Bcbot4, and Bcbot5) were exclusively induced during tomato infection, indicating differential regulation of this phytotoxin-associated pathway between the analyzed datasets. Moreover, among upregulated fungal DEGs in tomato, 180 and 196 genes encoded secreted enzymes at 23 and 47 hpi, respectively (Table S14). These DEGs included enzymes involved in the degradation of cutin, cell-wall components, carbohydrates, mannan, and plant cell wall–oligosaccharide side chains, as well as hydrolases, peptidases, and several virulence factors. Among them, 89 were candidate secreted effectors (80 apoplastic and nine cytoplasmic). When comparing the *B. cinerea* secretome between the two datasets, 135 DEGs encoding secreted enzymes were upregulated during infection in both plants, while 27 were exclusively induced in potato (Figure 7C, Table S15). These commonly upregulated DEGs encoded cutinases, glucanases, cellulases, polygalacturonases, xylanases, pectin lyases, pectate lyases, galactosidases, arabinofuranosidase, carboxylic ester hydrolases, glucosidases, and other enzymes capable of degrading various plant cell components such as cutin, carbohydrates, cellulose, hemicellulose, pectin, and oligosaccharide side chains. Furthermore, genes encoding virulence factors (Bcnep1, Bcnep2, Bcmp1, and CFEM domain-containing proteins), peptidases, and hydrolases were commonly induced. Among the DEGs induced only in potato, six were potential apoplastic effectors (including uncharacterized proteins and a chitinase), and one was a cytoplasmic effector (uncharacterized protein). Additional potato-specific secreted proteins included α-1,2-mannosidase, F5/8 type C domain-containing protein, Bcgod2, and several uncharacterized proteins. Conversely, during the infection in tomato, 115 fungal DEGs encoded secreted enzymes detected exclusively in this dataset, including 38 potential apoplastic effectors (such as pectinesterases, Bcpg1, Bcpg2, Bcpg5, pectate lyase, and Amb all domain-containing protein) and five cytoplasmic effectors. To facilitate comparison between the potato–*B. cinerea* and tomato–*B. cinerea* interactions, the main differences in fungal transcriptional profiles observed between the two datasets are summarized in Table 1.

### 2.7. Validation of RNA-Seq Data by qRT-PCR

**Figure 8:**
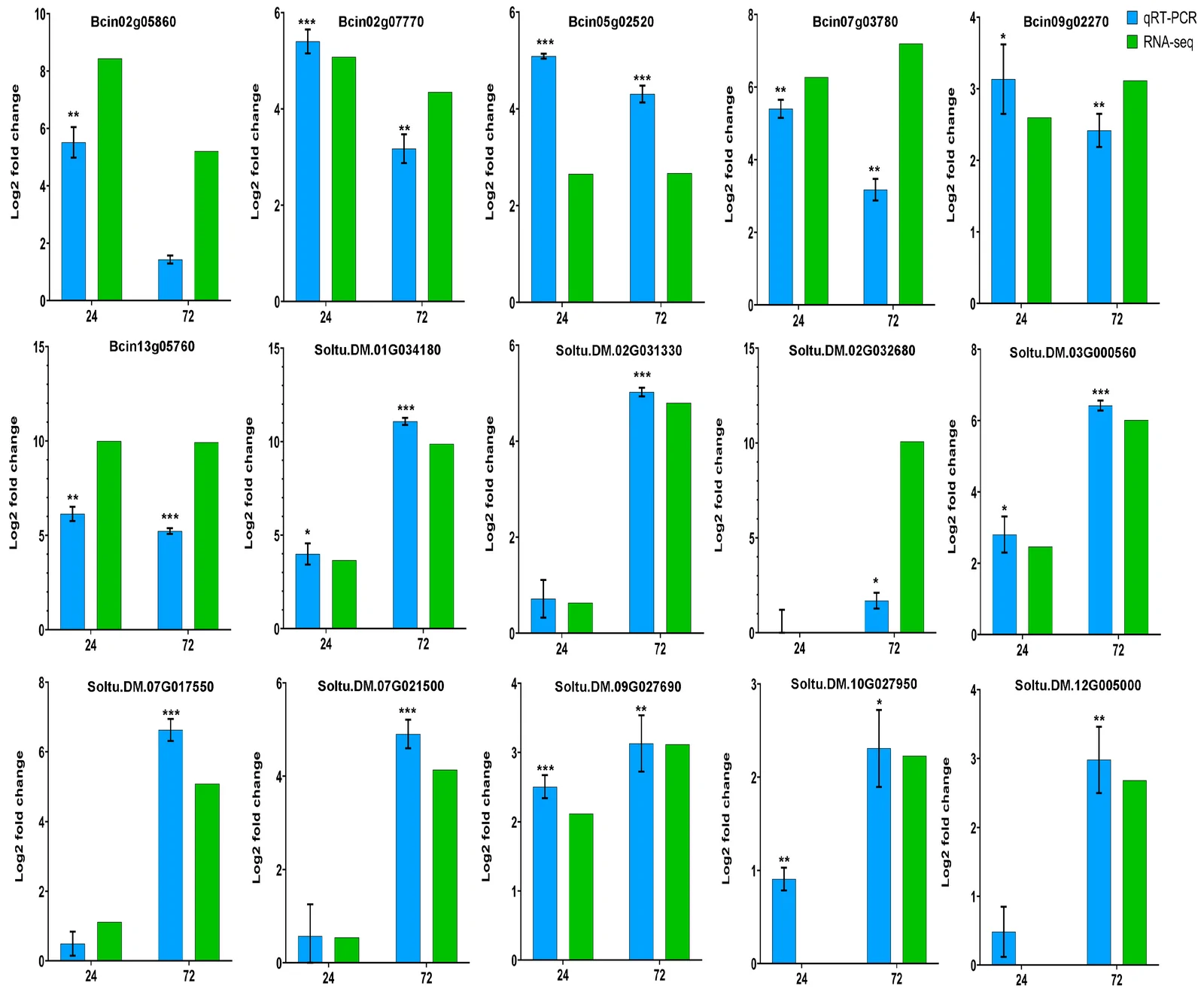
qRT-PCR validation of selected DEGs identified by RNA-seq. Relative expression levels of fifteen genes randomly selected from *B. cinerea* and potato DEGs during infection. Gene expression was normalized using a ubiquitin gene for *B. cinerea* and an elongation factor gene for potato. RNA-seq expression levels are presented as log_2_ fold change, whereas qRT-PCR expression levels were calculated using the 2^−ΔΔCt^ method and represented as log_2_(2^−ΔΔCt^)**.** Values represent means ± SD of three independent biological replicates. Asterisks indicate statistically significant differences compared with the corresponding control (Student’s *t*-test; * *p* < 0.05, ** *p* < 0.01; *** *p* < 0.005).

To validate the reliability of the RNA-seq data, the expression of 15 selected DEGs was analyzed by qRT-PCR. These genes were chosen to represent different expression patterns observed in the transcriptome analysis across infection time points. As illustrated in Figure 8, qRT-PCR analysis showed expression patterns consistent with those detected by RNA-seq, including gene induction at 24 or 72 hpi, as well as the absence of significant induction relative to the control in other cases. RNA-seq expression levels are presented as log_2_ fold change, whereas qRT-PCR expression levels were calculated using the 2^−ΔΔCt^ method and represented as log_2_(2^−ΔΔCt^) to facilitate comparison between the two approaches. Overall, the qRT-PCR results were consistent with the RNA-seq expression trends, supporting the reliability of the transcriptomic data generated in this study.

## 3. Discussion

In the present work, we investigated the interaction between potato plants and fungus *B. cinerea*, focusing on the characterization of plant and fungal gene expression profiles during infection. To place these responses in the context of previously published transcriptomic data from another solanaceous host, we also compared our data with publicly available RNA-seq datasets from *B. cinerea*-infected tomato [16]. Transcriptional changes in potato during *B. cinerea* infection reflected the activation of fungal virulence and colonization processes and plant pathogen perception and defense responses. Because the potato and tomato transcriptomic datasets were generated independently using different fungal isolates, inoculum concentrations, sampling time points, and experimental conditions, differences in transcriptional profiles may reflect both differences in infection stage and other experimental variables, in addition to potential host-associated effects. Despite these limitations, both datasets showed induction of genes associated with fungal virulence and plant defense responses, suggesting that common biological processes may be activated during the interaction between *B. cinerea* and solanaceous hosts.

Our findings show that during *B. cinerea* perception, potato modulates the expression of genes encoding PRRs and NLRs, with upregulation of multiple receptor families involved in pathogen and damage perception, including LRR-RLKs, CRKs, lectin-RLKs, WAK-RLKs, and LysM-RLKs, as well as disease-resistance proteins, consistent with activation of pathogen perception and immune-related pathways. Similar induction patterns have been reported in potato infected with necrotrophic fungus *Alternaria solani* [21]. Increased WAK-RLK expression has also been observed during potato infection by *Pectobacterium carotovorum* spp. *brasiliense* [22], consistent with their role in sensing cell-wall damage. Overexpression of WAK1 in *Arabidopsis thaliana* enhanced resistance to *B. cinerea* [23]. Potato contains more than 100 lectin-RLK genes, several of which are induced during interactions with *Fusarium* and *Phytophthora infestans* [24]. LysM-RLKs recognize N-acetylglucosamine-containing ligands, including chitin and peptidoglycan, and are involved in defense responses against phytopathogens [25]. Potato also induced *StLysM-RLK05* upon infection with *P. infestans* and *Streptomyces scabies*, with higher expression in moderately *S. scabies*-resistant potato lines [26]. In *A. thaliana*, LYM2, the homolog of a rice LysM RLP, is essential for defense against *B. cinerea* [27]. Furthermore, several potato CRKs are rapidly induced in response to treatments with bacterial virulence factors of *Pectobacterium wasabiae* and short oligogalacturonides (OGs), suggesting a possible role in the perception of this bacterium and DAMPs [28].

Comparison of tomato RNA-seq data at similar but not identical infection stages [16] with our potato dataset revealed consistent transcriptional profiles among PRR- and NLR-encoding genes, suggesting that both datasets show the activation of immune-related responses associated with perception of *B. cinerea* and/or cell-wall-derived molecules. The difference in sampling time should be considered when interpreting the comparative analysis, as the 23 hpi and 24 hpi samples were collected at closely spaced time points, whereas the 47 hpi and 72 hpi potato samples were collected at more distinct stages of infection and may therefore reflect differences in fungal colonization and host transcriptional responses. Both datasets also showed strong induction of genes involved in calcium signaling and MAPK pathways, which have been previously associated with *B. cinerea* responses [29,30]. ROS-associated responses were detected in both datasets, including genes involved in ROS production and detoxification. In potato, *RbohB* is stimulated by *P. infestans* elicitors and regulated by calcium-independent kinases [31], while tomato *RbohB* is required for resistance to *B. cinerea* [32]. Consistent with ROS production, both species induced oxidative stress-related genes involved in ROS detoxification. Finally, several transcription-factor families (including AP2, WRKY, MYB, HLH, and GRAS) were among the most represented DEGs in both hosts during *B. cinerea* infection. Many of these transcription-factor genes also exhibit differential expression in response to infections with *P. infestans*, *Ralstonia solanacearum*, and *Potato virus Y* [33], highlighting their central roles in Solanaceae immunity. Overall, these observations are consistent with the activation of shared immune-associated transcriptional responses in the two datasets.

*B. cinerea* infection in potato induces genes related to JA and ET. Comparable activation of JA- and ET-related genes was also observed in the tomato analysis, indicating that these hormonal pathways are transcriptionally responsive during infection in both hosts. Previous studies have demonstrated the relevance of JA and oxylipin pathways in response to *B. cinerea*. In tomato, LOX overexpression enhances resistance, whereas OPR3 silencing increases susceptibility [34,35]. In addition to JA biosynthesis, LOX enzymes catalyze oxylipin production, a group of oxygenated fatty acids involved in defense against pathogens and insects [36]. The induction of α-DOX1 genes only in *B. cinerea*-inoculated potato plants suggests differences in the transcriptional regulation of oxylipin-mediated responses, as α-dioxygenases contribute to the production of oxylipins involved in the regulation of cell death and antimicrobial defense responses [37,38]. ET signaling also plays a key role in *B. cinerea* resistance. In *A. thaliana*, ETRs and ERFs such as ERF1 are critical regulators of disease progression [39,40], and synergistic interactions between JA and ET pathways are required for effective defense [41]. In contrast, potato plants infected with *A. solani* exhibit reduced expression of JA and ET biosynthetic genes [21], highlighting pathogen-specific hormonal regulation. SA-related genes were also modulated during *B. cinerea* infection, with both species showing induction of PAL, a key enzyme involved in phenylpropanoid metabolism and SA biosynthesis [42], together with genes encoding SGT1. The role of SGT1 in disease resistance is not clear, since its overexpression in *A. thaliana* increases susceptibility to *Pseudomonas syringae*, while in rice, its silencing reduces chemically induced resistance [43,44]. Interestingly, NPR1, a central regulator of SA signaling [45], showed contrasting transcriptional patterns between potato and tomato, suggesting potential differences in SA–JA–ET regulatory dynamics during infection. In potato, NPR1 overexpression enhances resistance to *R. solanacearum* and increases the activity of defense-related enzymes [46]. In contrast, NPR1 knockout in tomato increases resistance to *B. cinerea* through enhanced JA–ET-mediated defenses [47]. Previous studies have shown that SA and JA signaling can act antagonistically during *B. cinerea* infection, with ET contributing to the modulation of both pathways [41]. Moreover, *B. cinerea* has been proposed to exploit SA-dependent signaling to attenuate effective JA–ET-mediated defenses and promote disease development in tomato [47]. In this context, the opposite regulation of NPR1 observed in potato and tomato suggests differences in the integration of SA–JA–ET signaling networks during infection. Repression of NPR1 in tomato may reflect a reduced contribution of SA-dependent signaling, potentially limiting the antagonistic effects of SA on JA–ET-associated defenses. Conversely, NPR1 induction in potato indicates a distinct hormonal regulatory response during infection. However, because these transcriptomic datasets were generated in independent studies and sampled at different stages of infection, the contrasting NPR1 expression patterns may also reflect differences in the temporal dynamics of hormone signaling rather than exclusively distinct defense strategies. Moreover, genes related to AUX, GA, and CK were predominantly downregulated in the tow analysis. ABA has been reported to negatively regulate defense responses against *P. infestans* in potato [48] and to increase susceptibility to *B. cinerea* in tomato by modulating SA-dependent signaling [49]. Therefore, the induction of ABA-related genes observed in potato should not necessarily be interpreted as a direct defense response. Instead, it may reflect a broader hormonal reprogramming during infection, whose biological significance requires further investigation. Overall, these observations indicate that hormone-associated transcriptional responses are extensively modulated during *B. cinerea* infection, while differences between the datasets should be interpreted in the context of their independent experimental designs.

Upon pathogen perception, plants activate multiple defense mechanisms, including the local and systemic induction of PR proteins [50]. The induction of PR proteins in both solanaceous hosts supports their important role in pathogen restriction during *B. cinerea* infection [39,51,52], a response also reported in *Vitis vinifera* [53]. The synthesis of antimicrobial secondary metabolites is a crucial component of plant defense [54]. In response to *B. cinerea*, both potato and tomato showed differential expression of genes associated with secondary metabolism, including terpenoid- and phenylpropanoid-associated pathways. However, differences in the transcriptional profiles were observed between potato and tomato. Potato preferentially induced genes associated with linalool-, germacrene-, and α-farnesene-related terpene biosynthetic pathways, whereas tomato preferentially induced genes associated with viridiflorene- and vetispiradiene-related terpene biosynthetic pathways. Terpenoid biosynthetic pathways are highly diversified in plants and contribute to defense against pathogens and herbivores through the production of specialized metabolites and volatile compounds [55,56]. The terpene-associated pathways identified here are consistent with the extensive diversification of terpene synthases previously described in tomato and other Solanaceae species [57]. Similar activation of specialized metabolism has been documented in potato during *A. solani* infection and in response to *P. infestans* [21,58]; *B. cinerea* also triggers antifungal metabolite production in grapevine [59]. Although transcriptomic analyses do not provide direct evidence of metabolite accumulation, the differential induction of terpene-associated biosynthetic genes suggests differences in the transcriptional regulation of specialized metabolism in the analyzed responses. Phenylpropanoid metabolism represents another important component of plant defense against pathogens. Consistently, fulvic acid-induced resistance to *B. cinerea* in grapevine involves activation of the phenylpropanoid pathway [60]. In our analysis, potato exhibited induction of coumarin-associated processes, whereas both species displayed activation of broader phenylpropanoid-related responses. Coumarins are phenylpropanoid-derived metabolites with antimicrobial activity against phytopathogens, including *B. cinerea* [61]. Beyond antimicrobial compound synthesis, the phenylpropanoid pathway contributes to lignin and phenolic polymer formation, reinforcing cell walls against pathogen degradation [62]. Thus, differences in specialized metabolite-associated transcriptional responses may reflect distinct regulatory strategies among Solanaceae hosts, although their contribution to resistance remains to be experimentally demonstrated. Potato similarly modulates genes related to cell-wall components upon *A. solani* infection [21]. Reinforcement and remodeling of the cell wall represent key defenses against pathogen invasion, and perturbations in cell-wall integrity can activate jasmonate-mediated immune responses [63]. HR-associated responses also differed between potato and tomato. The HR is another defense mechanism triggered at infection sites to restrain pathogen spread, being less effective against necrotrophic and hemibiotrophic pathogens. However, HR induction could be beneficial during necrotroph infection [64]. We detected contrasting regulation of HR-related processes: potato downregulated HR at 72 hpi, whereas tomato upregulated it at the corresponding time point of 47 hpi. These differences may reflect distinct regulation of cell-death-associated responses during infection; however, they should be interpreted cautiously, given the independent experimental conditions and sampling times.

To understand *B. cinerea* colonization in potato, we analyzed fungal gene expression at 24 and 72 hpi and compared it with tomato [16]. Fungal differential expression in both datasets was determined using RNA-seq libraries from *B. cinerea* grown on PDA as the reference condition. This in vitro reference was selected to provide a standardized baseline for fungal gene expression, whereas fungal material recovered from infected host tissue would already reflect transcriptional responses associated with host contact and infection. Comparative analyses suggest that *B. cinerea* activates common infection-associated processes in solanaceous hosts, while differences were observed in the expression of genes associated with secreted enzymes, effectors, and toxin-related functions. During infection, the fungus activates a coordinated set of extracellular processes required for host colonization, including degradation of plant structural components and adaptation to the host environment. *B. cinerea* secretes cutinases and lipases to breach the plant surface [11] and can penetrate directly through the cuticle using short germination tubes or through wounds and stomata [65]. The induction of multiple secreted hydrolases and proteases in both datasets is consistent with the central role of extracellular degradation during fungal invasion. Together, these processes likely contribute to nutrient acquisition and tissue colonization by facilitating the breakdown of plant structural barriers [65,66,67,68,69].

During penetration, *B. cinerea* promotes ROS accumulation, contributing to tissue colonization and the formation of primary lesions [11]. Several fungal enzymes may contribute to ROS generation during infection. One such enzyme, superoxide dismutase (BcSOD1), is expressed during cuticle penetration, and its deletion has been shown to reduce virulence in various hosts [11]. In our study, BcSOD1 was upregulated at 72 hpi in potato, whereas no differential expression was detected in tomato at the analyzed sampling time points. Together with the induction of ROS-associated genes, including oxidoreductases involved in oxidative metabolism [70], these observations suggest that fungal oxidative metabolism is tightly regulated during infection. Concurrently, plants produce ROS as a defense mechanism, emphasizing the importance of redox systems in maintaining oxidative homeostasis in fungi [71]. Accordingly, activation of fungal detoxification mechanisms likely contributes to fungal adaptation to the oxidative environment encountered during host colonization [72].

*B. cinerea* induce cell death in the host plant through production of OA, phytotoxic metabolites, and host-selective toxins (HSTs) [11]. Oxalic acid facilitates *B. cinerea* infection by increasing polygalacturonase activity, inhibiting plant defense enzymes, suppressing plant oxidative burst, and affecting stomatal closure, among other mechanisms [72]. The induction of OA-associated genes during potato infection, together with previous evidence showing that increased OA accumulation promotes *B. cinerea* infection in tomato [73], supports the relevance of oxalic acid metabolism during colonization of solanaceous hosts. *B. cinerea* produces two families of non-specific phytotoxins inducing chlorosis and cell collapse: i) botryane-type sesquiterpenes (mainly botrydial and dihydrobotridial) and ii) polyketides (including botcinic acid) [74]. Unlike OA-associated functions, genes related to botrydial and botcinic acid biosynthesis showed contrasting transcriptional patterns between the potato and tomato datasets. These contrasting patterns suggest differences in the transcriptional regulation of toxin-associated secondary metabolism during infection, although their biological significance may depend on the specific infection context.

The *B. cinerea* genome encodes several phytotoxic secreted proteins known as cell-death-inducing proteins (CDIPs), including BcNEP1 and BcNEP2, which trigger necrosis and ethylene production in host plants [72,75]. Genes encoding both proteins were expressed during infection of potato and tomato, consistent with the ability of *B. cinerea* to promote oxidative burst and hypersensitive-like responses to facilitate host colonization [76]. In addition to secreted toxins, fungal pathogens rely on membrane transporters to tolerate toxic compounds produced by plants [77]. These transporters mainly belong to the ATP-binding cassette (ABC) and major facilitator superfamily (MFS) families, with the *B. cinerea* genome encoding an expanded repertoire of MFS transporters [78]. The induction of MFS transporter genes during infection may contribute to fungal detoxification and adaptation to the host environment.

## 4. Materials and Methods

### 4.1. Biological Material and Treatments

*S. tuberosum* cv. Bintje axenic in vitro plants were originally obtained from the Instituto Nacional de Investigaciones Agropecuarias, Las Brujas, Uruguay. Plants were axenically micropropagated and grown in vitro as previously described [79]. Briefly, three plants per jar (90 mm × 125 mm) were axenically grown in vitro on 50 mL of MS2% agar medium (MS 4.3 g/L, Sucrose 20 g/L, MES 0.5 g/L, pH 5.7, Agar 9 g/L) [80] for 4 weeks at 22 °C under a photoperiod 16 h light/8 h dark (100–150 μmol m^−2^ s^−1^). A *B. cinerea* strain isolated from lemon plants [81] was cultivated on potato dextrose agar (PDA) (Sigma-Aldrich, St. Louis, MO, USA) at 22 °C. The fourth, fifth, and sixth leaves from the plant apex were infected by inoculating 3 µL of a *B. cinerea* water-spore solution (1 × 10^6^ spores/mL, approximately 3000 spores per leaf) into a slight wound on the leaf tissue made by the tip of an automatic micropipette to prevent droplet roll-off. Water-treated leaves were used as the inoculation control. Samples were collected for RNAseq analysis at two time points: 24 and 72 h post inoculation (hpi). For each time and treatment, three independent biological replicates were frozen in liquid nitrogen and stored at −80 °C. *B. cinerea* tissues were stained with 0.1% solophenyl flavine 7GFE 500 according to Ponce de Leon et al. [82] and visualized with an Olympus IX-81 inverted epifluorescence microscope (Shinjuku-ku, Japan) using DPController Software (version 1.1.1.65). Pictures were taken at 24, 48 and 72 hpi.

### 4.2. RNA Extraction, cDNA Library Construction and Sequencing

An RNeasy Plant Mini Kit (Qiagen, Hilden, Germany) was used to extract total RNA from the frozen samples with an added DNase treatment on column, according to the manufacturer’s protocol. RNA quality control, library preparation, and sequencing were performed at Macrogen Inc. (Seoul, Republic of Korea). RNA concentration and integrity were assessed using an Agilent 2100 bioanalyzer (Agilent Technologies, Santa Clara, CA, USA). cDNA libraries were prepared for paired-end sequence using a TruSeq Stranded Total RNA LT Sample Prep Kit (Plant), following the TruSeq Stranded Total RNA Sample Prep Guide. Three biological replicates, each containing three treated plants, were generated from each treatment, resulting in a total of 12 libraries. Sequencing of 151 bp paired-end libraries was performed on the Illumina platform (Illumina, San Diego, CA, USA) by Macrogen Inc. (Seoul, Republic of Korea).

### 4.3. S. tuberosum RNA-Seq Data Analysis

Sequence quality was assessed using FastQC software (https://www.bioinformatics.babraham.ac.uk/projects/fastqc; version 0.11.9, accessed on 1 February 2022), followed by trimming with Trimmomatic software (version 0.32) to obtain clean reads [83]. All Truseq3 adapters were trimmed, and SLIDINGWINDOW:4:15 HEADCROP:12 MINLEN:50 parameters were set additionally to default options. HISAT2 v2.1.0 [84] was used to align clean reads from all libraries to *S. tuberosum* reference genome DM v6.1 (https://spuddb.uga.edu/, accessed on 1 February 2022) with the DM_1-3_516_R44_potato.v6.1.hc_gene_models.gff3 file used as reference for genome annotation. Samtools view software v1.9 was used to obtain BAM files, which were sorted by name with Samtools Sort software v2.0.3 [85]. Read counting was performed with FeatureCounts software v2.0.0 [86], using the same reference genome (DM v6.1), applying parameters to count fragments rather than reads, allowing reads to contribute to multiple features, and including multi-mapping reads/fragments, in addition to default settings. Differential expression analyses between treatments (*B. cinerea* vs. water-treated control) for each time point were performed using the EdgeR package [87]. Library sizes were normalized using the TMM method. Differential expression was assessed using the quasi-likelihood F-test (QLF). *p*-values were adjusted using the Benjamini–Hochberg method [88]. Differentially expressed genes (DEGs) were identified using a false discovery rate (FDR) ≤ 0.05 and log2 fold change ≥2 or ≤−2.

### 4.4. B. cinerea RNA-Seq Data Analysis

To identify fungal DEGs, cDNA libraries from *B. cinerea* mycelium grown on PDA medium for 12 days were used as a control. These cultures were grown under the same conditions used for spore production in the infection assays and were used as a standardized in vitro reference for comparison with fungal gene expression during host colonization. RNA-seq data analysis was performed using the same pipeline described in Section 4.3 (*S. tuberosum* RNA-seq data analysis), with some modifications. *B. cinerea* isolate B05.10 (ASM14353v1) (http://fungi.ensembl.org/Botrytis_cinerea/Info/Index, accessed on 1 February 2022) was used as the reference genome, with the Botrytis_cinerea.ASM83294v1.55.gff3 file as reference for genome annotation. Identified fungal DEGs were compared with previously published *B. cinerea* secretomes [19,20]. Potential effectors were predicted using EffectorP 3.0 (http://effectorp.csiro.au/, accessed on 1 October 2022). In addition, secreted proteins involved in pathogenicity were identified using the PHI-base v4.15 database (http://www.phi-base.org/, accessed on 1 October 2022).

### 4.5. Solanum Lycopersicum–B. cinerea Interaction RNA-Seq Data Analysis

RNA-seq sequence reads from *S. lycopersicum* plants infected with *B. cinerea* [16] were obtained from NCBI. These reads correspond to plant leaves inoculated with 5 µL of *B. cinerea* strain B05.10 spore solution (2 × 10^5^ spores/mL, 6000 spores per leaf). We selected the 23 hpi (SRR12676681, SRR12676679, and SRR12676678) and 47 hpi (SRR12676684, SRR12676685, and SRR12676686) time points, which were the closest timepoints for comparison with our study. To identify *S. lycopersicum* DEGs, RNA-seq data analysis was performed using a pipeline similar to that described for *S. tuberosum*, with some modifications: TruSeq3 adapters were specified for single-end reads in Trimmomatic software v0.32 to obtain clean reads and used *S. lycopersyium* ITAG4.0 as the reference genome (https://phytozome-next.jgi.doe.gov/info/Slycopersicum_ITAG4_0, accessed on 1 October 2022), with the Slycopersicum_691_ITAG4.0.gene.gff3 file as reference for genome annotation. As controls, 0 hpi samples (SRR12676688, SRR12676689, and SRR12669316) were used. To identify fungal DEGs, RNA-seq data analysis was performed using a pipeline similar to that described for *S. tuberosum*, with some modifications: TruSeq3 set for single-ended in Trimmomatic software v0.32 to obtain clean reads and *B. cinerea* isolate B05.10 used (ASM83294v1) (https://jun2026-fungi.ensembl.org/Botrytis_cinerea/Info/Index, accessed on 1 October 2022) with the Botrytis_cinerea.ASM83294v1.55.gff3 file as reference for genome annotation. The same cDNA libraries from *B. cinerea* grown on PDA described above were used as the fungal control condition. Fungal DEGs encoding secreted proteins were identified using the same methodology described in Section 4.4.

### 4.6. Gene Ontology Enrichment Analysis and Orthologous Gene Identification

Gene Ontology (GO) annotations of *S. tuberosum* and *S. lycopersicum* DEGs were obtained from the Phytozome v13 platform (http://www.phytozome.net/, accessed on 1 October 2022), and functional enrichment analysis was performed using the GOEnrichment tool available through the Galaxy platform (https://usegalaxy.org/, accessed on 1 October 2022). GO and functional annotations of *B. cinerea* DEGs were assigned using G: profiler (https://biit.cs.ut.ee/gprofiler/, accessed on 1 October 2022). GO terms with an adjusted *p*-value < 0.05 were considered for our analysis. Orthologous genes were identified between *S. tuberosum* and *S. lycopersicum* DEGs using OrthoFinder v. 2.5.5 [89] with default parameters. The corresponding protein sequences were obtained from the Phytozome v13 platform (http://www.phytozome.net/, accessed on 1 December 2024).

### 4.7. Validation of Differentially Expressed Genes by RT-qPCR

To validate the RNA-seq results, a subset of 15 DEGs was randomly selected, including genes from potato and *B. cinerea*. Total RNA samples used for RNA-seq were treated with DNase I to remove residual genomic DNA, and first-strand cDNA was synthesized using the RevertAid Reverse Transcriptase kit (Thermo Scientific, Waltham, MA, USA), following the manufacturer’s instructions. Quantitative real-time PCR (qRT-PCR) reactions were performed in a final volume of 20 µL using the QuantiTect SYBR Green PCR Kit (Qiagen, Hilden, Germany). Gene-specific primers were designed for each selected DEG (Table S1). For normalization, a potato elongation factor gene was used as the internal reference for potato genes, while a ubiquitin gene was used as the reference for *B. cinerea* genes. All reactions were carried out using three independent biological replicates. Relative gene expression levels were calculated using the 2^−ΔΔCt^ method [90].

## 5. Conclusions

Our comparative transcriptomic analysis reveals a conserved defense program in potato and tomato in response to *B. cinerea*, involving pathogen perception, calcium and MAPK signaling, ROS modulation, hormone signaling, and activation of defense-related and secondary metabolic pathways. At the same time, the datasets revealed differences in the regulation of immune and secondary metabolism biosynthesis, as well as in fungal virulence-associated functions, including toxin and effector production, which may reflect host-, pathogen-, or condition-dependent variation. The conserved responses identified here point to core mechanisms underlying *B. cinerea* infection in Solanaceae, while the contrasting transcriptional patterns suggest differences in host responses and fungal adaptation. Despite differences in the experimental datasets, this comparative analysis provides a framework for identifying conserved and distinct components of potato– and tomato–*B. cinerea* interactions and to guide future studies under standardized experimental conditions.

## Figures and Tables

**Table 1:** Summary of transcriptional differences observed between potato and tomato *B. cinerea* infection datasets.

Feature	Potato	Tomato
JA/ET biosynthesis genes	OPR3 and SAMs (induced)	No specific induction detected
SA signaling (NPR1)	Upregulated	Downregulated
HR-related pathways	Downregulated	Upregulated
Secondary metabolism genes	Linalool-, germacrene-, α-farnesene-, and coumarin-related genes (induced)	Viridiflorene-, vetispiradiene-, and costunolide-related genes (induced)
Fungal toxins–botrydial	Bcbot1–4 (repressed)	Bcbot1–5 (induced)
Fungal toxins–botcinic acid	Bcboa2–7, 9, 12–13, and 16–17 (repressed)	Bcboa17 (induced)
Fungal ROS-related genes	BcSOD1 and oxidoreductases (induced)	Partially induced

## Data Availability

All raw RNA-Seq read data were deposited in the National Center for Biotechnology Information (NCBI) Short Read Archive (http://www.ncbi.nlm.nih.gov/sra/) under BioProject accession code PRJNA1163727.

## Supplementary Materials

The following supporting information can be downloaded at: https://www.mdpi.com/article/10.3390/plants15182815/s1. Table S1: List of primers used in this study; Table S2: Summary of *S. tuberosum* mapped reads of the RNA-Seq libraries; Table S3: List of *S. tuberosum* differentially expressed genes (DEGs) during *B. cinerea* infection at 24 and 72 hpi; Table S4: *S. tuberosum* enriched gene ontology (GO) terms (over-representation) for biological processes (BPs), molecular function (MF), and cell compartment (CC) at 24 and 72 hpi; Table S5: List of *S. lycopersicum* differentially expressed genes (DEGs) during *B. cinerea* infection at 23 and 48 hpi; Table S6: Orthogroups inferred with OrthoFinder from DEGs in *S. tuberosum* and *S. lycopersicum* during *B. cinerea* infection; Table S7: *S. lycopersicum* enriched gene ontology (GO) terms (over-representation) for biological processes (BPs), molecular function (MF), and cell compartment (CC) at 23 and 48 hpi; Table S8: Summary mapped reads of *B. cinerea* in the RNA-Seq libraries; Table S9: List of *B. cinerea* differentially expressed genes (DEGs) during *S. tuberosum* infection at 24 and 72 hpi; Table S10: *B. cinerea* enriched gene ontology (GO) terms (over-representation) for biological processes (BPs), molecular function (MF), and cell compartment (CC) at 24 and 72 hpi; Table S11: List of *B. cinerea* upregulated DEGs encoding secreted proteins during infection of *S. tuberosum* at 24 and 72 hpi; Table S12: List of *B. cinerea* differentially expressed genes (DEGs) during *S. lycopersicum* infection at 23 and 47 hpi; Table S13: List of *B. cinerea* DEGs exclusively upregulated during infection of *S. tuberosum* or *S. lycopersicum*.; Table S14: List of *B. cinerea* upregulated DEGs encoding secreted proteins during infection of *S. lycopersicum* at 23 and 47 hpi; Table S15: List of upregulated DEGs encoding secreted proteins during *S. tuberosum* and *S. lycopersicum* infection; Figure S1: *S. tuberosum* and *S. lycopersicum* DEGs related to isoquinoline alkaloid biosynthesis identified in response to *B. cinerea* infection. Schematic representation of the isoquinoline alkaloid biosynthesis pathway. Color coding, arrow styles, and time-point designations are as described in Figure 3.

## Abbreviations

The following abbreviations are used in this manuscript:

4CL	4-coumarate-CoA ligase
ACX	Acyl-CoA oxidase
ACO	Aminocyclopropanecarboxylate oxidase
ACS	1-aminocyclopropane-1-carboxylate synthase
AFS1	α-farnesene synthase
AOC	Primary-amine oxidase
AOS	Hydroperoxide dehydratase
BDH	Borneol dehydrogenase
C3H	Coumarate 3-hydroxylase
C4H	Cinnamic acid 4-hydroxylase
CAD	Cinnamyl-alcohol dehydrogenase
CCOMT	Caffeate O-methyltransferase
CCR	Cinnamoyl-CoA reductase
CHI	Chalcone isomerase
CHS	Chalcone synthase
CM	Chorismate mutase
CODM	Codeine 3-O-demethylase
COMT	Caffeoyl/CoA-3-O-methyltransferase
CS	Chorismate synthase
CYP71BL2	Costunolide synthase
DOX	α-dioxygenase
EAH	5-epiaristolochene 1,3-dihydroxylase
EAS	5-epiaristolochene synthase
EBF1/2	EIN3-binding F-box protein
EIN3	Ethylene-insensitive protein 3
EPSP	3-phosphoshikimate 1-carboxyvinyltransferase
ERF1	Ethylene-responsive transcription factor 1
ETR	Ethylene receptor
F3H	Flavanone 3-hydroxylase
F3′5′H	Flavonoid 3′,5′-hydroxylase
F3′H	Flavonoid 3′-hydroxylase
F7GT	Flavanone 7-O-beta-glucosyltransferase
FLS	Flavonol synthase
GAO	Germacrene A hydroxylase
GBS	Germacrene B synthase
GDS	Germacrene D synthase
G10H	Geraniol 8-hydroxylase
HCT	Shikimate O-hydroxycinnamoyltransferase
HGD	8-hydroxygeraniol dehydrogenase
HPO	Premnaspirodiene oxygenase
HVS	Vetispiradiene synthase
IPD	Isopiperitenol dehydrogenase
JAZ	Jasmonate ZIM domain-containing protein
JMT	Jasmonate O-methyltransferase
KAT	Acetyl-CoA C-acyltransferase
LIS	S-linalool synthase
LMT	Loganate O-methyltransferase
LOX	Lipoxygenase
MFS	Menthofuran synthase
NCS	Norcoclaurine synthase
NMD	Neomenthol dehydrogenase
NPR1	Nonexpressor of pathogenesis-related genes 1
OPCL1	8:0 CoA ligase 1
OPR3	12-oxophytodienoic acid reductase
PAL	Phenylalanine ammonia-lyase
PAT	Prephenate aminotransferase
POD	Peroxidase
PR	Pathogenesis-related proteins
RLS	R-linalool synthase
SAMS	S-adenosylmethionine synthetase
SD	Shikimate dehydrogenase
SK	Shikimate kinase
SLS	Secologanin synthase
SR	Salutaridine reductase
T6ODM	Thebaine 6-O-demethylase
THBO	Tetrahydroberberine oxidase
TPS31	Viridiflorene synthase
tyrAa	Arogenate dehydrogenase
UGT8	7-deoxyloganetic acid glucosyltransferase
ZSS1	α-humulene synthase
